# Intrarenal Arterial Transplantation of Dexmedetomidine Preconditioning Adipose Stem-Cell-Derived Microvesicles Confers Further Therapeutic Potential to Attenuate Renal Ischemia/Reperfusion Injury through miR-122-5p/Erythropoietin/Apoptosis Axis

**DOI:** 10.3390/antiox11091702

**Published:** 2022-08-30

**Authors:** Yu-Hsuan Cheng, Kuo-Hsin Chen, Yi-Ting Sung, Chih-Ching Yang, Chiang-Ting Chien

**Affiliations:** 1 Department of Life Science, School of Life Science, College of Science, National Taiwan Normal University, No. 88, Section 4, Tingzhou Road, Taipei 11677, Taiwan; 80943003s@ntnu.edu.tw (Y.-H.C.); stacylukedog@gmail.com (Y.-T.S.); 2 Department of Surgery, Division of General Surgery, Far-Eastern Memorial Hospital, New Taipei City 22056, Taiwan; chen.kuohsin@gmail.com; 3 Department of Electrical Engineering, Yuan Ze University, Taoyuan City 32003, Taiwan; 4 Office of Public Relation of Ministry of Health and Welfare, No. 488, Section 6, Zhongxiao E. Rd., Nangang District, Taipei 115204, Taiwan; 5 MacKay Junior College of Medicine, Nursing and Management, New Taipei City 11260, Taiwan

**Keywords:** adipose-derived mesenchymal stem cells, apoptosis, dexmedetomidine preconditioning, erythropoietin, ischemia/reperfusion, microvesicles, miR-122-5p

## Abstract

Intravenous adipose mesenchymal stem cells (ADSCs) attenuate renal ischemia/reperfusion (IR) injury but with major drawbacks, including the lack of a specific homing effect after systemic infusion, cell trapping in the lung, and early cell death in the damaged microenvironment. We examined whether intrarenal arterial transplantation of dexmedetomidine (DEX) preconditioning ADSC-derived microvesicles (DEX-MVs) could promote further therapeutic potential to reduce renal IR injury. We evaluated the effect of DEX-MVs on NRK-52E cells migration, hypoxia/reoxygenation (H/R)-induced cell death, and reactive oxygen species (ROS) amount and renal IR model in rats. IR was established by bilateral 45 min ischemia followed by 4 h reperfusion. Intrarenal MVs or DEX-MVs were administered prior to ischemia. Renal oxidative stress, hemodynamics and function, western blot, immunohistochemistry, and tubular injury scores were determined. The miR-122-5p expression in kidneys was analyzed using microarrays and quantitative RT-PCR and its action target was predicted by TargetScan. DEX-MVs were more efficient than MVs to increase migration capability and to further decrease H/R-induced cell death and ROS level in NRK-52E cells. Consistently, DEX-MVs were better than MV in increasing CD44 expression, improving IR-depressed renal hemodynamics and renal erythropoietin expression, inhibiting IR-enhanced renal ROS level, tubular injury score, miR-122-5p expression, pNF-κB expression, Bax/caspase 3/poly(ADP-ribose) polymerase (PARP)-mediated apoptosis, blood urea nitrogen, and creatinine levels. The use of NRK-52E cells confirmed that miR-122-5p mimic via inhibiting erythropoietin expression exacerbated Bax-mediated apoptosis, whereas miR-122-5p inhibitor via upregulating erythropoietin and Bcl-2 expression reduced apoptosis. In summary, intrarenal arterial DEX-MV conferred further therapeutic potential to reduce renal IR injury through the miR-122-5p/erythropoietin/apoptosis axis.

## 1. Introduction

Renal ischemia/reperfusion (IR) injury can be caused by cardiac surgery, renal vascular obstruction, and kidney transplantation, mainly leading to acute kidney injury, which is complicated by a lack of effective preventative and therapeutic strategies. Overproduction of reactive oxygen species (ROS) can evoke oxidative stress and trigger the translocation of nuclear factor-kappa B (NF-κB) to the nucleus to activate several inflammatory cytokines and adhesion molecules, leading to inflammation and apoptosis [1]. The increased ROS by IR can enhance oxidative stress signaling, especially Bax/Bcl-2/caspase 3/poly-(ADP-ribose)-polymerase (PARP)-mediated apoptotic cell death [2], consequently leading to acute renal injury.

Mesenchymal stem cells can be obtained from umbilical cord, placenta, bone marrow, and adipose tissue [3]. Among them, adipose-derived stem cells (ADSCs) are abundant and easy to access vs. stem cells from other sources. The cell-based mesenchymal stem cells have been applied in the clinical trial but with some limitations and challenges, including the cell trapping in the pulmonary microcirculation with clotting risk, the extremely low survival rate of transplanted stem cells in the ischemic tissue, and the unguided homing effect. It is mentioned that ADSCs through paracrine mechanisms provide excellent self-renewal and proliferation ability through high levels of growth factors, cytokines, microvesicles (MVs) or exosomes carrying noncoding RNAs such as microRNAs (miRNAs) in the microenvironment or conditioned medium [4,5]. MiRNAs are small noncoding RNAs with 22~25 nucleotides in eukaryotes that can be contained in MVs and transferred to neighboring or distant cells, and can modulate the function of recipient cells. MiRNAs combined with the 3′-terminal nontranslation region (3′-UTR) of mRNA of the target gene to degrade mRNA or inhibit mRNA translation, to regulate the expression of the target gene to participate in cell survival and proliferation and regulation of various biological processes such as apoptosis, oxidative stress, and other biological processes [6,7,8,9]. MV and exosomes derived from stem cells can facilitate intercellular communication by transferring proteins and miRNAs [6]. The contribution of miRNAs to the pathogenesis of several diseases has been demonstrated previously [7,8,9]. The decreased miR-122 attenuated cisplatin-induced acute kidney injury [7] and cerebral IR injury [9]. Thus, we suggest that ADSC-derived MVs may ameliorate renal IR injury through the action of miRNAs.

A previous report using exendin-4-assisted ADSC therapy was superior to either one alone for preserving kidney function in acute kidney IR injury, suggesting that pharmacological preconditioning ADSCs further enhanced renal protection [10]. Intravenous anesthetics such as dexmedetomidine (DEX) could bind with the α_2_ adrenoceptor to exert sedative effects in various clinical situations in intensive care units. In addition, DEX with anti-inflammatory and renoprotective effects [11] and co-treatment with mesenchymal stem cells can further attenuate hepatic IR injury [12]. To date, there is no literature exploring whether DEX-treated ADSC could elevate the efficacy against renal IR injury through DEX preconditioning ADSC-MVs. In the present study, we preconditioned the ADSCs with DEX to explore the DEX-MVs’ effect on migration activity, hypoxia/reoxygenation (H/R)-induced renal proximal tubular cells injury, and IR-induced acute kidney injury. 

## 2. Methods and Materials

### 2.1. Animals and ADSC Preparation

Female Wistar rats (200 to 250 g) at the age of 10 weeks were purchased from BioLASCO Taiwan Co., Ltd. (Ilan, Taiwan) and housed at the Experimental Animal Center, National Taiwan Normal University, at a constant temperature and with a consistent light cycle (light from 07:00 to 18:00 O’clock). Food and water were provided ad libitum. All surgical and experimental procedures were approved by National Taiwan Normal University Institutional Animal Care and Use Committee (Approval number 105035) and were in accordance with the guidelines of the National Science Council of Republic of China (NSC 1997).

ADSC were isolated from subcutaneous flank adipose tissue of 10 anesthetized rats as previously described [13,14]. Briefly, under urethane anesthesia, isolated flank adipose tissue was collected into centrifuge tubes under the sterile condition. The adipose tissue was stirred to aspirate off saline and oil phases and washed 3 to 5 times with phosphate buffer saline (PBS), and the lower phase was discarded until clear. The final upper phase was collected and collagenase was added and incubated for 1 to 4 h at 37 °C on a shaker. After incubation, 10% fetal bovine serum (FBS; Invitrogen) was added into the tube to neutralize collagenase. The fluid in the tube containing digested fat was centrifuged at 800× *g* for 10 min. Then, we aspirated the supernatant composed of floating adipocytes, lipids and liquid, and left stromal vascular fraction (SVF) pellet. We used 160 mM NH_4_Cl to suspend SVF pellet, which was then incubated for 10 min at room temperature. After incubation, the pellet was centrifuged at 400× *g* for 10 min at room temperature. The final pelleted fraction of mononuclear cells was then resuspended in DMEM (Dulbecco’s Modified Eagle’s Medium; Sigma-Aldrich, Darmstadt, Germany) supplemented with 40% FBS, penicillin-streptomycin, and 10 ng/mL EGF (Invitrogen) incubated on Petri dish overnight to select for adherent cells. The remaining cells and debris were aspirated off on the next day and the plate was washed with PBS. ADSCs were maintained on DMEM low glucose supplemented with 10% FBS, 1% penicillin-streptomycin and L-glutamine at 5% CO_2_ 37 °C. Primary ADSC were seeded into a T75 culture flask and grown in complete culture medium DMEM, supplemented with 10% FBS, 100 units/mL penicillin, 100 μg/mL streptomycin, and 2 mM L-glutamine, and changed every 2 days.

### 2.2. Identification of ADSCs by Surface Marker

Isolated ADSCs were seeded into a T75 culture flask for 24 h, then fixed by 4% paraformaldehyde for 30 min and incubated with positive surface marker, FITC mouse anti-rat CD90 (BD Pharmingen, La Jolla, CA, USA), anti-rat CD29 (BD Pharmingen), and a negative marker, FITC mouse anti-rat CD31 (BD Pharmingen), anti-rat CD45 (BD Pharmingen) for 30 min. ADSCs were washed by phosphate-buffered saline (PBS) three times and analyzed by flow cytometry (BD FACSAria III; BD Biosciences, San Jose, CA, USA). A purified ADSC was characterized for highly positive expression of CD44, CD29, and CD90 and negative CD31 and CD45 expression [15].

### 2.3. Purification of MVs

The ADSCs were pretreated with a selective agonist of the α2-adrenergic receptor and imidazoline receptors dexmedetomidine (DEX) [16,17] for 24 h. ADSCs or DEX-treated ADSCs were seeded in 6-well plates for 24 h before being collected in 1.5 mL tube. The tubes with ADSCs were placed in a thermomixer (ThermoMixer^®^ C, Eppendorf, Germany) for 30 min at 300 rpm room temperature. The isolation procedure of MV was described previously [18,19]. ADSCs were centrifuged 10 min at 300× *g*, 4 °C to discard dead cells and cell debris. The supernatant was collected and centrifuged three times, which were 10 min 2000× *g*, 30 min 10,000× *g*, and 30 min 10,000× *g* at 4 °C. The final pellets filled with isolated MVs were suspended with PBS and recentrifuged for 30 min at 10,000× *g*, 4 °C to remove contaminating proteins. We used a scanning electron microscope (JEOL-6500F, JEOL, Tokyo, Japan) to characterize the morphology and size of the MV. The images were analyzed using the ImageJ software (National Institutes of Health, NIH, Bethesda, MD, USA). One previous study indicated that intravenously injected stem cells were trapped in lung tissue and did not reach the damaged kidney tissue [20]. To prevent ADSCs being trapped in lung tissue and to confer high protective efficacy to the damaged kidney, intrarenal arterial MVs or DEX-MVs were administered at the dose of 15 μg/mL in saline. The isolated MV marker was characterized with CD44 by Western blot analysis.

### 2.4. Cell Culture

Renal proximal tubular cell, NRK-52E cells (American Tissue Type Culture Collection, Manassas, VA, USA) were cultured in high-glucose Dulbecco’s modified Eagle’s medium (DMEM) (Gibco, Grand Island, NY, USA) supplemented with 10% fetal bovine serum (FBS) (Gibco, Grand Island, NY, USA) and 1% penicillin-streptomycin (Mediatech, Mooresville, NC, USA) in a humidified atmosphere with 5% CO_2_ at 37 °C. When treated with MV or DEX-MV, cells with 1 × 10^5^ cells/well were cultured in serum-free medium for 2 h previously.

### 2.5. Cell Viability by MTT Assay

The MTT (3-(4,5-Dimethylthiazol-2-yl)-2,5-diphenyltetrazolium bromide) (Sigma, St. Louis, MO, USA) assay was utilized as a method to determine the cell vitality. NRK-52E cells with 1 × 10^5^ cells/well were incubated with MV or DEX-MV for 1.5 h in the incubator with MTT. Then, the treated NRK-52E cells were exposed to hypoxia (5% CO_2_, 1% O_2_ and 94% N_2_) for 4 h, then to reoxygenation (5% CO_2_, 21% O_2_ and 74% N_2_) for 2 h. The optical density value was determined and read by an ELISA reader (MRX Microplate Reader, Dynex Technologies, Inc., Chantilly, VA, USA) at 570 nm.

### 2.6. Wound Healing Assay

Wound healing assay was performed to detect the cell migration of NRK-52E cells, which were seeded into a 6-well plate with 1 × 10^5^ cells/well for 12 h, and then incubated with MV or DEX-MV for 24 h and grown until 90% confluence. Then, NRK-52E cells created an artificial wound by disposable 10 μL pipette tips. Detaching NRK-52E cells were washed out by serum-free condition medium three times after being scratched and cultured with serum-free condition medium. The NRK-52E cells’ migration was imaged under an inverted microscope and photographed at the times of 0 and 24 h of culturing.

### 2.7. Extraction of RNA for miRNA Assay

Extraction of total RNA, small RNA (fewer than 200 nucleotides), and large RNA from MV, DEX-MV, control kidney, IR kidney, and MV- and DEX-MV-treated kidney was performed using a SeraMir^TM^ Exosome RNA Amplification Kit (System bioscience) according to the manufacturer’s instructions. The following miRNA was used: rno-miR-122-5p MIMAT0000827 UGGAGUGUGACAAUGGUGUUUG.

### 2.8. Grouping and Renal IR Induction

Twenty-four female rats (n = 6 in each group) were divided into control (CON), ischemia/reperfusion (IR), IR with ADSC-derived MV treatment (IR+MV) and IR with DEX-MV treatment (IR + DEXMV) groups. The rats with or without renal IR (n = 6 in each group) were anesthetized with intraperitoneal urethane (1.2 g/kg). The trachea was exposed via a midline cervical incision and intubated. For the induction of ischemia, the bilateral renal arteries were clamped for 45 min with a small vascular clamp. Sham-operated animals underwent similar operative procedures without occlusion of the renal arteries. Reperfusion was initiated by the removal of the clamp for 4 h. After IR insults, arterial blood and urine were collected for renal functional determination [blood urea nitrogen (BUN) and creatinine]. After sacrifice with intravenous KCl, these two kidneys were resected and divided into two parts. One part was stored in 10% neutral buffered formalin for immunohistochemistry and in situ pathologic assay, and the other was quickly frozen in liquid nitrogen and stored at −70 °C for protein isolation.

### 2.9. Tracking of Intrarenal Arterial Administered MV and DEX MV in Rat Kidneys

To determine the MV or DEX MV expression in the kidney, we injected MV or DEX MV containing a green fluorescent protein (GFP) into the kidney and examined the GFP expression in rat kidneys one hour later. The renal sections were investigated under a fluorescent microscope for the detection of fluorescence in the kidneys. Western blot with primary antibodies against CD44 (MCA643GA, Serotec, Kidlington, UK) was examined in the kidney homogenates for quantification of CD44 expression, which is a positive ADSC surface biomarker [15] in these kidneys.

### 2.10. In Vivo and In Vitro Chemiluminescence (CL) Recording for Measurement of Reactive Oxygen Species (ROS) Activity

The technique for superoxide anion measurement from the kidney surface in vivo in response to IR was performed by the intrarenal arterial administration of a superoxide anion probe, 2-Methyl-6-(4-methoxyphenyl)-3,7-dihydroimidazo-[1,2-a]-pyrazin-3-one-hydrochloride (MCLA) (0.2 mg/mL/h, TCI-Ace, Tokyo Kasei Kogyo Co., Ltd., Tokyo, Japan) and determined with a Chemiluminescence Analyzing System (CLD-110, Tohoku Electronic In. Co., Sendai, Japan) [1].

The in vitro ROS measurement from the NRK-52E cells with 1 × 10^5^ cells/well was performed with an ultrasensitive chemiluminescence analyzer, as described previously [1]. Immediately before CL measurement, 0.1 mL of phosphate-buffered saline (pH 7.4) was added to 0.2 mL of cell cultures. The CL was measured in a completely dark chamber of the Chemiluminescence Analyzing System. After 100 s background level determination, 1.0 mL of 0.1 mM lucigenin in phosphate-buffered saline (pH 7.4) was injected into the sample. The CL was monitored continuously for an additional 600 s. The total amount of CL was calculated by integrating of the area under the curve and subtracting it from the background level. The assay was performed in duplicate for each sample and was expressed as CL counts/10 s. The mean ± SEM of the CL level of each sample was calculated.

### 2.11. Renal Microcirculation Determination

To examine the MV and DEX-MV effect on IR-evoked renal hemodynamics change, a full-field laser perfusion imager (MoorFLPI, Moor Instruments Ltd., Devon, UK) was used to continuously quantitate the renal microcirculatory blood flow intensity during the periods of baseline, ischemia, and reperfusion, as described previously [21].

### 2.12. Renal Arterial Blood Flow Determination

To examine MV and DEX-MV effect on IR induced renal hemodynamics alteration continuously, including 60 min of baseline, 45 min of ischemia, and 4 h of reperfusion periods, we measured the renal arterial blood flow by placing the renal artery on a blood flow probe and connected to a transonic flow meter (T420, Transonic System Inc., Ithaca, NY, USA) and recorded on a Power Lab system (ML870 PowerLab 8/30, ADInstruments Pty Ltd., Castle Hill, NSW, Australia).

### 2.13. Western Blot

Twenty µg of protein was electrophoresed as follows: The total proteins were homogenized with a prechilled mortar and pestle in extraction buffer consisting of 10 mM Tris-HCl (pH 7.6), 140 mM NaCl, 1 mM phenylmethyl sulfonyl fluoride, 1% Nonidet P-40, 0.5% deoxycholate, 2% β-mercaptoethanol, 10 µg/mL pepstatin A, and 10 µg/mL aprotinin. The homogenate was centrifuged at 12,000 rpm for 12 min at 4 °C, the supernatant was collected, and the protein concentrations were determined by BioRad Protein Assay (BioRad Laboratories). Antibodies raised against the monoclonal mouse anti-human PARP (Promega, Madison, WI, USA), polyclonal rabbit anti-rat caspase 3 (Chemicon, CA, USA), primary antibody Bcl-2 (Santa Cruz, Dallas, TX, USA), Bax (Abcam, Cambridge, UK), rabbit polyclonal EPO (Abcam, Cambridge, UK), and monoclonal mouse anti-mouse β-actin (Sigma, Saint Louis, MI, USA) were used at 1:400. The membranes were incubated with goat anti-rabbit or anti-mouse IgG secondary antibodies conjugated to horseradish peroxidase (Biolegend, San Diego, CA, USA) for 1 h, washed in TBST, and imaged with Image Quant^TM^ LAS 4000 (GE, Boston, MA, USA).

### 2.14. Histologic Studies

Kidney samples were fixed in 4% paraformaldehyde and embedded in paraffin. Five-micrometer sections were stained with hematoxylin and eosin (H&E). Twenty sections in each kidney were randomly selected at ×200 magnification, and the degree of renal damage was scored using the scoring system for renal injury, as described previously [20]. The percentage of tubular injury parameters containing epithelial flattening, tubular atrophy, tubular dilatation, and brush border loss was estimated by a five-point scale. The degree of injury was graded on a scale from 0 to 4, where 0 = normal; 1 = mild, involvement of less than 25% of the cortex; 2 = moderate, involvement of 25–50% of the cortex; 3 = severe, involvement of 50–75% of the cortex; and 4 = extensive damage, involving more than 75% of the cortex.

### 2.15. Immunohistochemistry

Five-micrometer tissue sections were deparaffinized in xylene, rehydrated in an ethanol series, and submitted to antigen retrieval step. The sodium citrate buffer (10 mM sodium citrate, 0.05% Tween 20, pH 6.0) was used for heat-induced epitope retrieval. After 15 min of antigen retrieval step, the sections were blocked for nonspecific binding with 5% bovine serum albumin (Sigma-Aldrich, St. Louis, MO, USA) for 1 h at room temperature and incubated with the primary antibodies for 18 h at 4°C. These sections were washed with PBS three times and then were incubated with primary antibodies, including rabbit anti-Caspase 3 (1:100; Chemicon), rabbit anti-PARP (1:100, Promega), and rabbit anti-NF-κB (1:100; R&D Systems, Minneapolis, MN, USA).

### 2.16. Immunofluorescence Stain

NRK-52E cells were plated at 1 × 10^5^ cells/well in a 96-well plate, and transfection was performed after 24 h incubation. The groups were as following (1) control with transfection reagent; (2) H/R treatment; (3) miR-122-5p mimics treatment; (4) H/R and miR-122-5p mimics treatment; (5) H/R and miR-122-5p inhibitor treatment; and (6) H/R and EPO treatment. MiR-122-5p mimic (5-AACGCCAUUAUCACACUAAAUA-3′) and inhibitor (5′-CAAACACCAUUGUCACACUCCA-3′) were purchased from PHALANX Biotech (Hsin-Chu City, Taiwan) and transfected with Lipofectamine 2000 (Invitrogen, Carlsbad, CA, USA) into cells 1 h before treatment with or without H/R injury. The samples were washed with DPBS three times, fixed with 4% paraformaldehyde, and treated with Triton X-100 on ice. The samples were incubated in blocking buffer for 1 h, and primary antibody Bcl-2 (Abcam, Cambridge, UK) and Bax (Novus Biologicals, CO, USA) were added overnight. The samples were then incubated with goat-anti-rabbit (Alexa Fluor 488) (Abacm) and goat-anti-mouse (Cy3) (Biolegend, CA, USA) for 1 h, and DAPI was added to identify nucleus. The immunofluorescence-stained samples were analyzed with a Zeiss LSM 880 with Airyscan (Zeiss, Oberkochen, Germany). Annexin-V-FITC apoptosis detection kit I (Becton Dickinson, Franklin Lakes, NJ, USA) was used to detect apoptotic cells. At the end of incubation, cells were stained with Annexin-V according to the manufacturer’s instructions. After staining procedure, cells were fixed with 4% paraformaldehyde for 20 min. Stained NRK-52E cells were photographed by using a fluorescence microscope (Olympus BX50, Tokyo, Japan).

### 2.17. Luciferase Reporter Assay

The binding sites of EPO and miR-122-5p were predicted using the Targetscan database (http://www.targetscan.org/vert_72/, accessed on 15 April 2021). After transfection, the cells were collected and lysed, followed by detection of luciferase activity with the help of Dual-Luciferase Reporter Assay System (Promega, Madison, WI, USA).

### 2.18. Statistical Analysis

All values were expressed as mean ± standard error mean (SEM). Differences within and among groups were evaluated by a one-way analysis of variance with a post hoc comparison. Differences were regarded as significant if *p* < 0.05 was adapted.

## 3. Results

### 3.1. Characteristics and Effect of MV and DEXMV on Cell Viability, Wound Healing Rate, and Antioxidant Activity

**Figure 1:**
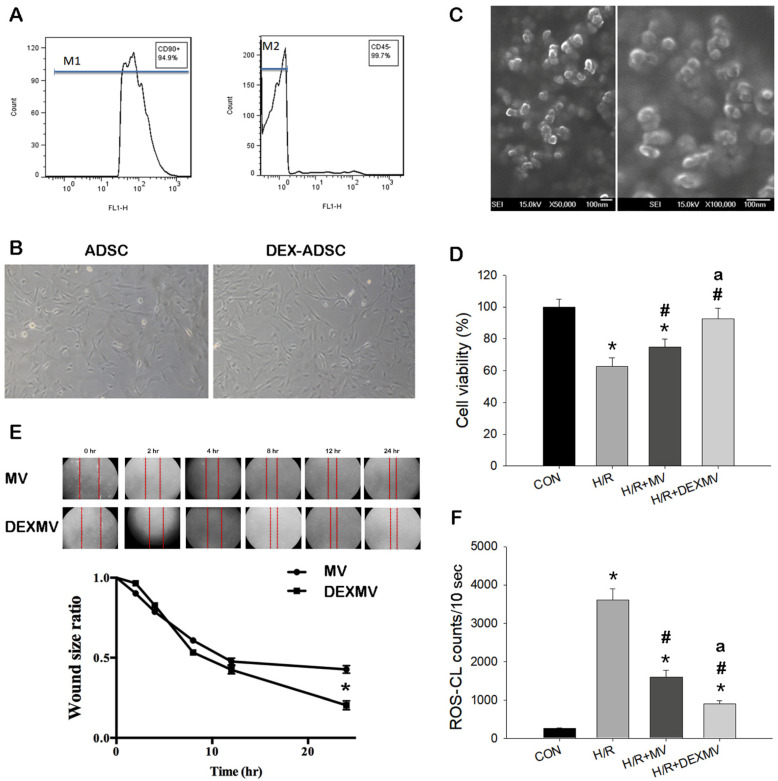
Identification of purified ADSC with CD90-positive characterization and CD45-negative characterization in the P4 ADSC by flow cytometry (**A**). The morphology in control ADSC (ADSC) and DEX-treated ADSC (DEX-ADSC) (Magnification Scale: 50 μm) in 80% confluency is similar in our result (**B**). Cells displayed fibroblast-like morphology (long and thin) under phase-contrast microscope. Nanoparticle size of MV is amplified 50,000 times (left) and 100,000 times (right), existing in ADSC culture medium (**C**). Effect of MVs and DEX-MVs on wound healing of NRK-52E cells in different time courses (**D**). DEX-MVs significantly decreased wound size ratio as compared to MVs. NRK-52E cells in response to H/R significantly decreased viability (**E**) and increased ROS (**F**) vs. CON. MVs or DEX-MVs significantly improved cell viability and reduced ROS counts vs. H/R group. DEX-MVs were more efficient than MVs in increase in cell viability and decrease in ROS counts. n = 6 in each experiment. ADSC, adipose stem cell; DEX, dexmedetomidine; MV, microvesicle; H/R, hypoxia/reoxygenation; ROS, reactive oxygen species. * *p* < 0.05 vs. CON. # *p* < 0.05 vs. H/R. a *p* < 0.05 vs. MV.

We first characterized the isolated ADSCs of cultured cells of the fourth passage by flow cytometry. Our data revealed that 94.9% of cells expressed CD90 positive expression and 99.7% expressed CD45 negative expression (Figure 1A). We compared the preconditioning effect of DEX to the ADSCs on morphologic analysis. The morphology of ADSC in control and DEX treatment displayed a similar shape in the ADSCs (Figure 1B), implying a nondetrimental effect of DEX on ADSC culture. We next characterized the particle size of ADSC-derived MVs under a scanning electron microscope and found that the MVs displayed a uniform morphology and size around 58.6 ± 6.1 nm (Figure 1C). We evaluated the effect of MVs or DEX-MVs in response to hypoxia-/reoxygenation-induced injury in NRK-52E cells by MTT assay. Our data found that H/R reduced NRK-52E cell viability and MVs or DEX-MVs restored the decreased cell viability (Figure 1D). Furthermore, DEX-MVs were more efficient than MVs in recovering cell viability. The scratch size for evaluating wound healing assay was demonstrated in different time courses between MV or DEX-MV groups (Figure 1E). DEX-MV treatment showed a higher migration capability compared to MV treatment. In addition, DEX-MVs acted more effectively than MV- and H/R-induced ROS amounts in NRK-52E cells (Figure 1F).

### 3.2. DEXMVs Were More Efficient Than MVs in Increasing CD44 Expression in Rat Kidneys

**Figure 2:**
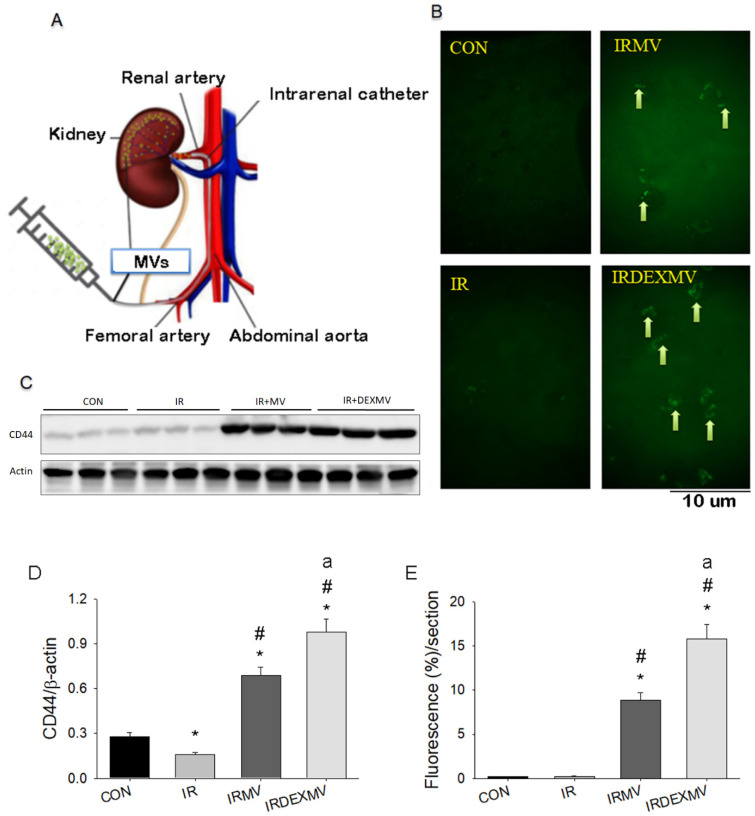
(**A**): The experimental protocol for intrarenal arterial injection of fluorescent MV to the kidney. (**B**): Detection of fluorescence expression in kidneys of rats treated with ADSC-derived MVs (n = 6). Representative fluorescent micrographs showing the expression of CD44 proteins in kidney sections of four groups of rats and sacrificed 1 h later. Original magnification: ×400. Arrows indicate fluorescent MVs. Six animals per groups were examined with similar results. (**C**): Western blot analysis of CD 44 expression of kidney homogenates in four groups of kidneys. (**D**): Statistical data of CD44 expression by Western blot (n = 3 each). (**E**): Statistical data of fluorescent MV expression of four groups of kidneys (n = 6 each). ADSC, adipose stem cell; MV, microvesicle; IR, ischemia/reperfusion; IRMV, ischemia/reperfusion with microvesicle. * *p* < 0.05 vs. CON rat. # *p* < 0.05 vs. IR rat. a *p* < 0.05 vs. IRMV rat.

We administered the PBS, MVs, or DEXMVs through the intrarenal arterial catheter from the femoral artery to the kidneys, as the graph demonstrates in Figure 2A. To ascertain the localization of MVs in the kidneys, we labeled the MVs with fluorescent dye GFP and identified their localizations in the treated kidneys. Our data showed that the fluorescent MVs indicated by the yellow arrow were found in IRMV and IRDEXMV kidneys but not in CON or IR kidneys (Figure 2B). The fluorescent intensity of MVs was stronger in IRDEXMV than that in IRMV kidneys (Figure 2E). We confirmed the CD44 expression, which was a specific surface marker of ADSC-derived MVs, in the kidneys by Western blot (Figure 2C). We found that CD44 expression was significantly enhanced in IRMV and IRDEXMV kidneys, whereas CD44 expression somewhat decreased in IR kidneys compared to CON kidneys (Figure 2D). Our result observed that the renal trafficking CD44 expression was much higher in IRDEXMV than that in IRMV kidneys (Figure 2D), suggesting a higher homing effect in DEXMVs.

### 3.3. MVs and DEXMVs Preserved Renal Microcirculation, Hemodynamics, and Function

**Figure 3:**
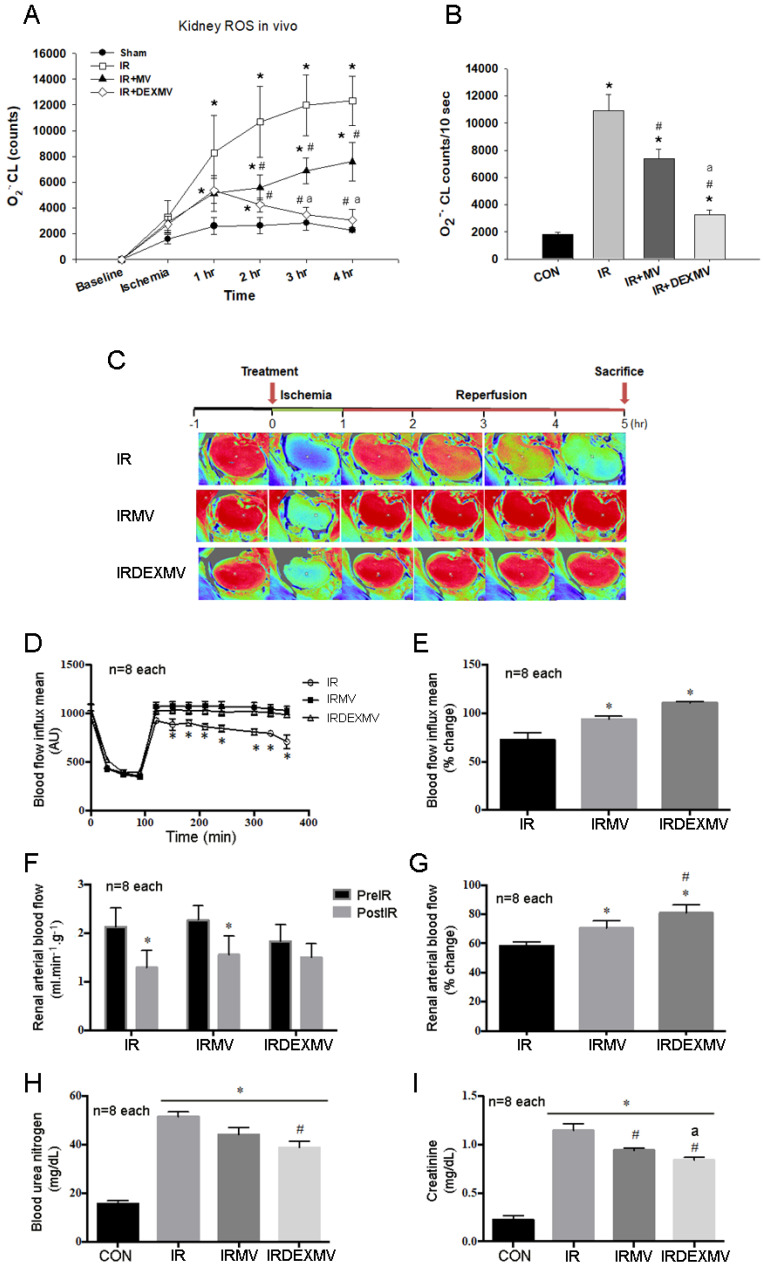
In response to renal IR injury, in vivo kidney ROS measurement was indicated in four groups of rats (n = 6 each) (**A**). The average ROS amount (n = 6 each) was displayed in (**B**). Renal microcirculation was determined with a moor image in three IR groups of rats (**C**). Renal IR significantly decreased renal microcirculation, as indicated by PU value in IR rats vs. IRMV and IRDEXMV treated rats (n = 6 each) (**D**). Renal PU significantly recovered towards the normal value within 10 min reperfusion in IRMV- and-IRDEXMV treated rats (n = 6 each). (**E**): The percentage change of blood flow influx mean is shown. Renal arterial blood flow is significantly decreased in IR and IRMV rats, but not in IRDEXMV rats (n = 6 each) (**F**). Percentage change of renal arterial blood flow is significantly increased in IRMV− and IRDEXMV−treated rats vs. IR rats within 10 min reperfusion (n = 6 each) (**G**). Renal IR significantly increases blood urea nitrogen (**H**) and creatinine (**I**) levels in IR−, IRMV−, and IRDEXMV− treated rats (n = 6 each). However, these two levels are significantly decreased in IRDEXMV vs. IR rats. * *p* < 0.05 vs. CON rat. MV, microvesicle; IR, ischemia/reperfusion; IRMV, ischemia/reperfusion with microvesicle; IRDEXMR, ischemia/reperfusion with dexmedetomidine preconditioned microvesicle; PU, perfusion unit; ROS, reactive oxygen species. # *p* < 0.05 vs. IR rat. a *p* < 0.05 vs. IRMV rat.

To determine the effect of MVs and DEXMVs on kidney ROS, renal microcirculation, renal arterial blood flow, and renal function, we compared these parameters in response to renal IR injury in the rats. As shown in Figure 3A, the in vivo kidney ROS amount was significantly increased in response to IR injury in IR, IR + MV, and IR + DEXMV groups. The IR-enhanced kidney ROS level was significantly depressed by MV or DEXMV treatment (Figure 3B). Our data further demonstrated that DEXMVs were more efficient than MVs to reduce kidney ROS value.

As displayed in Figure 3C, in the baseline period, the kidney displayed a red color with high PUs in three groups of rats. Forty-five-minute ischemia severely decreased the renal microcirculation and PUs in IR-, IRMV-, and IRDEXMV-treated kidneys. In contrast, during the reperfusion stage, there was significant recovery of renal microcirculation in the IRMV and IRDEXMV groups vs. the IR group (Figure 3D). Depressed renal microcirculation by ischemia was significantly recovered towards normal values within 10 min reperfusion in the IRMV and IRDEXMV groups vs. the IR group. The percentage change of blood flow influx mean was displayed in the order of IRDEXMV > IRMV > IR rats (Figure 3E). We further confirmed renal blood flow response by a transonic flowmeter. Our data observed that post-IR renal arterial blood flow was significantly decreased in IR and IRMV rats, but not in IRDEXMV-treated rats (Figure 3F). A significant increase in percentage change of renal arterial blood flow within 10 min reperfusion was found in IRMV and IRDEXMV vs. IR kidneys (Figure 3G). The percentage change of renal arterial blood flow was significantly increased in IRDEXMV as compared to IRMV rats, implying a faster recovery of renal arterial blood flow with DEXMV treatment.

Renal IR significantly increased blood urea nitrogen (Figure 3H) and creatinine (Figure 3I) levels in IR-, IRMV-, and IRDEXMV-treated rats. However, these parameters were significantly decreased in IRDEXMV vs. IR rats, implying that IRDEXMV treatment was more efficient in improving renal function than IRMV.

### 3.4. MVs and DEX-MVs Reduced IR-Induced Inflammation and Apoptosis

**Figure 4:**
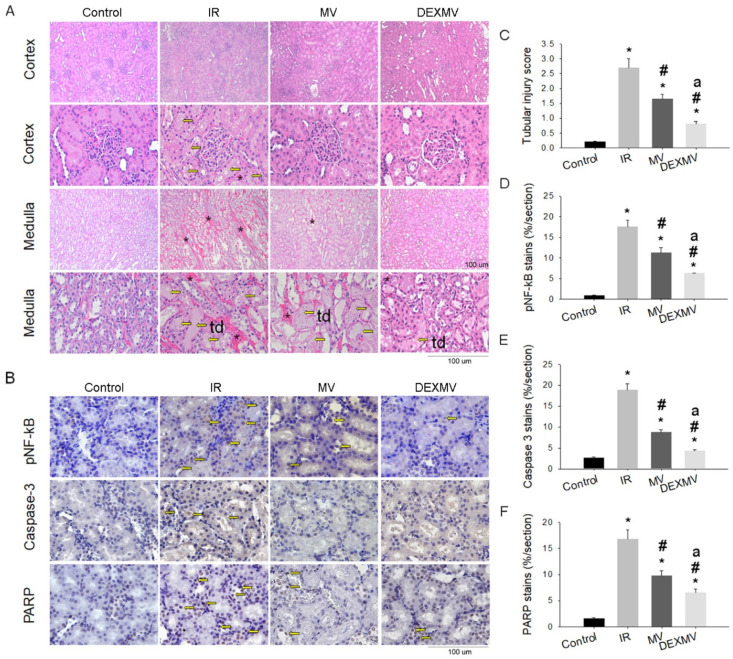
Effect of MVs or DEXMVs on pathologic parameters in renal cortex and medulla by H&E stain (**A**), pNF-κB, Caspase-3, and PARP stain (**B**) in CON, IR, MV, and DEXMV groups. The statistical data of tubular injury score (**C**), p-NF-κB expression (**D**), Caspase-3 expression, (**E**) and PARP expression (**F**) are indicated. Data are expressed as mean ± SEM in each group (n = 6) using single values. * *p* < 0.05 compared with CON group. td: tubular dilation. Yellow arrows in (**A**) indicate tubular dilation (td). Asterisks in (**A**) indicate erythrocyte accumulation. Yellows in (**B**) indicate each brown stain. MV, microvesicle; IR, ischemia/reperfusion; IRMV, ischemia/reperfusion with microvesicle; IRDEXMR, ischemia/reperfusion with dexmedetomidine preconditioned microvesicle; PARP, poly-(ADP-ribose)-polymerase; SEM, standard error mean. # *p* < 0.05 compared with IR group. a *p* < 0.05 vs. MV group.

We found that a significantly increased tubular injury score, including epithelial flattening, tubular atrophy, tubular dilatation and brush border and erythrocyte accumulation, was demonstrated in IR kidneys (Figure 4A), whereas the tubular injury score was significantly reduced in IRMV and IRDEXMV kidneys (Figure 4C). Furthermore, IRDEXMV decreased the tubular injury score further than IRMV did.

We explored the effect of MVs on renal IR-evoked inflammatory transcription factor (pNF-κB), apoptosis-related caspase-3, and PARP expression in four groups of rats. Compared to CON, the expression of pNF-κB, c-caspase-3, and PARP expression was all enhanced in IR kidneys (Figure 4B), whereas the expression in pNF-κB (Figure 4D), cleaved caspase-3 (Figure 4E), and PARP (Figure 4F) was significantly downregulated in IRMV and IRDEXMV kidneys. Our data also implied that DEX preconditioning ADSC MVs were more effective in the IRDEXMV than in the IRMV group at attenuating IR-induced inflammation and apoptosis.

### 3.5. MiR-122-5p Was the Highest-Expression miRNA in IR Kidneys

**Figure 5:**
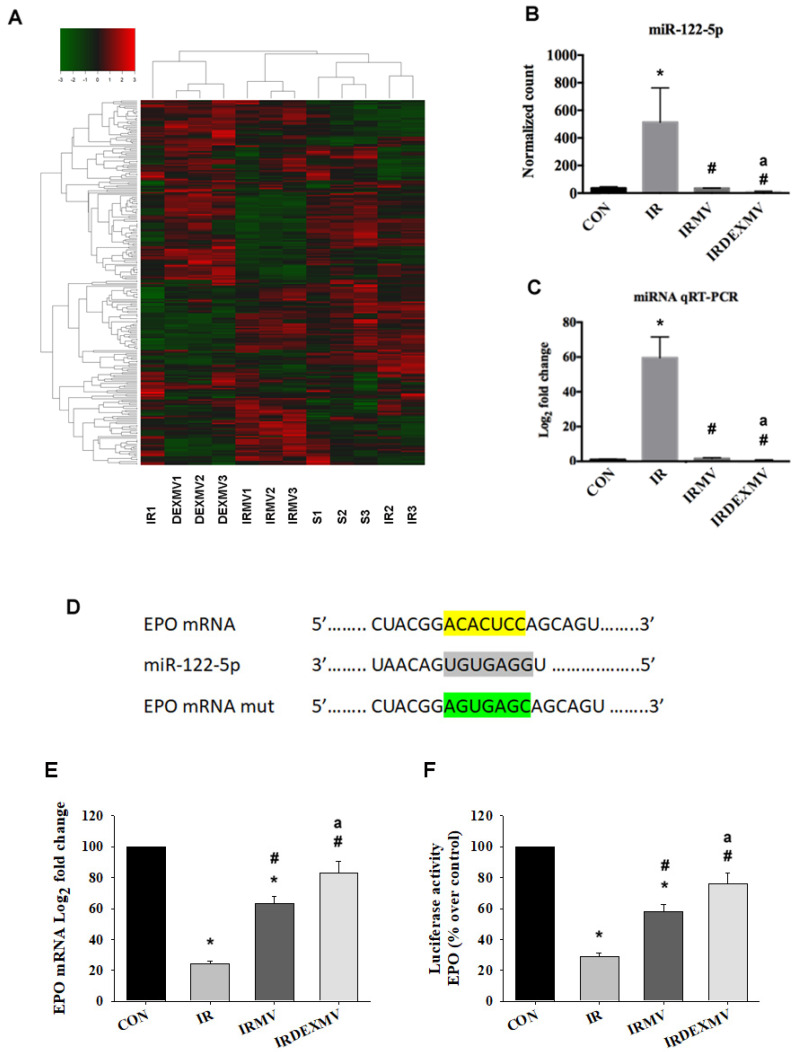
miRNA expression in four groups of kidneys. (**A**) The heat map shows the expression level of whole−kidney miRNAs in CON (S1, S2, S3), IR (IR1, IR2, IR3), IRMV (IRMV1, IRMV2, IRMV3), and IRDEXMV (DEXMV1, DEXMV2, DEXMV3) (n = 3 each). Each column represents a sample and each row represents a miRNA in the graph. The expression ratio is represented by color ranges from green (low) to red (high), as indicated by the scale bar. (**B**) The highest relative expression level of miR-122-5p in microarray is analyzed and displayed in these four groups. (**C**) Relative expression of miR-122-5p in kidney is detected by quantitative RT−PCR. (**D**) To understand the mechanism whereby miR-122-5p contributes to IR, by using online databases (TargetScan), we have identified a conserved putative miR-122-5p-targeting site in the EPO mRNA. (**E**) Erythropoietin (EPO) mRNA levels from four groups of kidneys were determined by quantitative RT−PCR. Relative fold-change values were normalized against GAPDH as endogenous control and expressed as fold changes over sham control kidney. (**F**) Luciferase activity of EPO in four groups of kidneys. Data are expressed as mean ± SEM in each group (n = 3) using single values. * *p* < 0.05 compared with CON group. # *p* < 0.05 compared with IR group. EPO, erythropoietin; MV, microvesicle; IR, ischemia/reperfusion; IRMV, ischemia/reperfusion with microvesicle; IRDEXMR, ischemia/reperfusion with dexmedetomidine preconditioned microvesicle; RT−PCR, real−time polymerase chain reaction; SEM, standard error mean. a *p* < 0.05 vs. IRMV group.

The differentially expressed miRNAs and the whole distribution of differentially expressed miRNAs were clustered and plotted with a heatmap (Figure 5A). Compared to CON, a significant upregulation of miR-122-5p expression levels was found in the IR group, while downregulation of miR-122-5p expression levels was found in the IRMV and IRDEXMV groups (*p* < 0.05, Figure 5B). We further noted that the miR-122-5p expression levels in IRDEXMV were significantly lower than those in the IRMV group. We confirmed the significant upregulation of miR-122-5p expression in IR kidneys vs. CON kidneys by quantitative real-time PCR (qRT-PCR) and significant downregulation of miR-122-5p in the IRMV and IRDEXMV groups (Figure 5C). The action site varication by TargetScan (Figure 5D), quantitative PCR (Figure 5E), and dual luciferase reporter gene assays (Figure 5F) were used to confirm that erythropoietin (EPO) was a target of miR-122-5p. Collectively, these findings indicated that IR increased miR-122-5p expression and decreased EPO expression as compared to the CON group. MV treatment significantly reduced miR-122-5p expression and increased EPO expression in the IRMV and IRDEXMV groups. A further downregulation of miR-122-5p expression and an upregulation of EPO expression were found in the IRDEXMV group as compared to the IRMV group.

### 3.6. MVs and DEX-MVs Decreased IR-Induced Apoptosis by Western Blot

**Figure 6:**
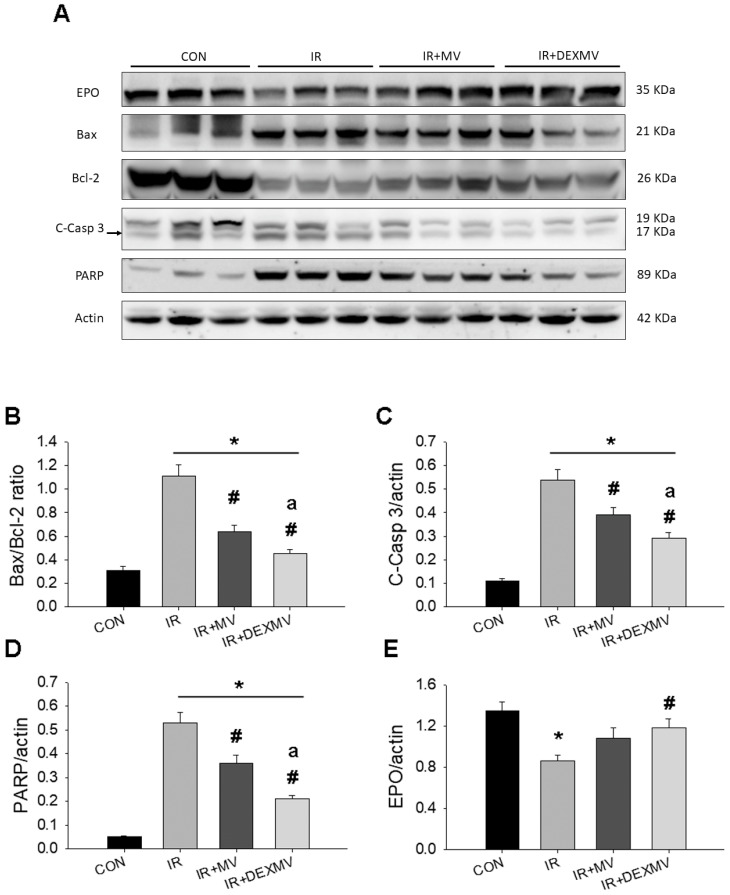
Effect of ADSC-derived MVs (IRMV) or DEX preconditioning ADSC-derived MVs (IRDEXMV) on Bax, Bcl-2, cleaved caspase-3 (c-caspase-3), PARP, and EPO expression in the four groups of kidneys. (**A**): The original data of Western blot. (**B**): Statistical data of the ratio of Bax/Bcl-2. (**C**): Statistical data of the ratio of C-Casp 3/actin. (**D**): Statistical data of the ratio of PARP/actin. (**E**): Statistical data of the ratio of EPO/actin. Data are expressed as mean ± SEM in each group (n = 3) using the single values. * *p* < 0.05 compared with CON group. ADSC, adipose stem cells; c-caspase-3, cleaved caspase-3; EPO, erythropoietin; MV, microvesicle; IR, ischemia/reperfusion; IRMV, ischemia/reperfusion with microvesicle; IRDEXMR, ischemia/reperfusion with dexmedetomidine preconditioned microvesicle; PARP, poly-(ADP-ribose)-polymerase; SEM: standard error mean. # *p* < 0.05 compared with IR group. a *p* < 0.05 compared with IR+MV group.

To verify the possible role of miR-122-5p-targeted gene EPO expression, we compared the expression of EPO and Bax/Bcl-2/C-caspase 3/PARP-mediated apoptotic signaling pathway in these four groups of kidneys by Western blot analysis. Figure 6A showed the typical Western blot of five proteins’ expression in four groups of rats. Compared to CON kidneys, the significantly upregulated expression of Bax, Bax/Bcl-2 ratio (Figure 6B), c-caspase-3 (Figure 6C), and PARP (Figure 6D) was indicated in IR kidneys, whereas the downregulated expression of Bcl-2 was demonstrated in IR kidneys (Figure 6A). When compared to IR kidneys, the significant decrease in Bax/Bcl-2 ratio, c-capase-3, and PARP expression was found in IRMV and IRDEXMV kidneys.

Because EPO could attenuate DNA fragmentation and prevent caspase-3 activation through upregulation of Bcl-xL [22], we also determined the renal EPO expression in response to IR injury. Renal EPO expression was significantly decreased in IR kidneys vs. CON kidneys, while the depressed EPO expression was partly recovered in IRMV- and IRDEXMV-treated kidneys (Figure 6E). As compared to IR kidneys, the degree of recovered renal EPO expression was significantly enhanced in IRDEXMV kidneys, and not in IRMV kidneys.

### 3.7. MiR-122-5p Inhibitor or EPO Attenuated H/R Induced Annexin V-Apoptosis through Upregulation of Bcl-2 and Downregulation of Bax in NRK-52E Cells by Immunofluorescence

**Figure 7:**
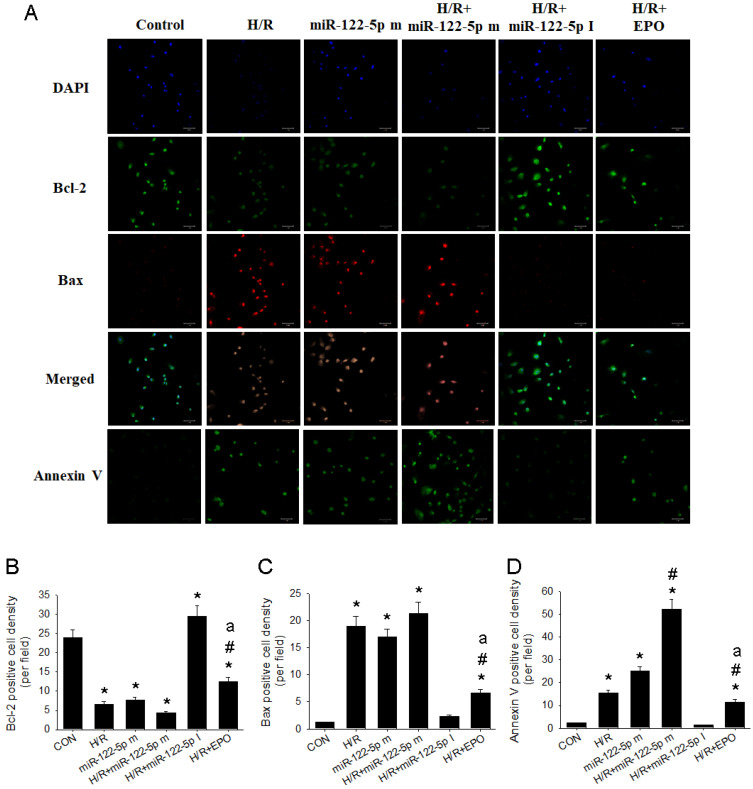
Effect of miR-122-5p mimics and inhibitor on H/R-induced Bcl-2, Bax, and apoptosis formation in the NRK-52E cells. (**A**): The immunofluorescence graphs of DAPI (blue), Bcl-2 (green), Bax (red), merged image, and Annexin V (green) in response to Scheme 2 positive cell density; (**B**): Bcl-2 positive cell density, (**C**): Bax positive cell density and (**D**): Annexin V positive cell density. Data are expressed as mean ± SEM (n = 3). * *p* < 0.05 compared with CON group. DAPI, 4’, 6-diamidino-2-phenylindole; EPO, erythropoietin; H/R, hypoxia/reoxygenation; SEM, standard error mean. # *p* < 0.05 compared with H/R group. a *p* < 0.05 vs. H/R+miR-122-5p m group.

To test the functional role of miR-122-5p and EPO on H/R injury in the renal proximal tubular cells, we transfected miR-122-5p mimics and inhibitor into NRK-52E cells and analyzed the expression level of Bcl-2-, Bax-, and Annexin V-mediated apoptosis by an immunofluorescence assay. As shown in Figure 7A, H/R markedly enhanced Bax and depressed Bcl-2 expression, subsequently leading to the increase in Annexin V-apoptosis expression in NRK-52E cells. MiR-122-5p mimics significantly decreased Bcl-2 expression (*p* < 0.05, Figure 7B), increased Bax expression (*p* < 0.05, Figure 7C), and promoted Annexin V-mediated apoptosis (*p* < 0.05, Figure 7D) in these cells. H/R plus miR-122-5p mimics seem to further decrease Bcl-2 expression and enhance Bax expression, resulting in a higher Annexin V-mediated apoptosis formation in the NRK-52E cells. The use of miR-122-5p inhibitor significantly decreased H/R injury by the upregulated Bcl-2 expression and the downregulated Bax expression, contributing to a lower Annexin V-apoptosis in NRK-52E cells. We verified the possible role of EPO on H/R injury of NRK-52E cells. Our data observed that EPO treatment significantly reduced H/R injury through the upregulation of Bcl-2 expression and downregulation of Bax expression and Annexin V-apoptosis formation.

### 3.8. MiR-122-5p Inhibitor or EPO Ameliorated H/R-Induced Apoptosis through the Upregulation of Bcl-2 and Downregulation of Bax in NRK-52E Cells by Western Blot

**Figure 8:**
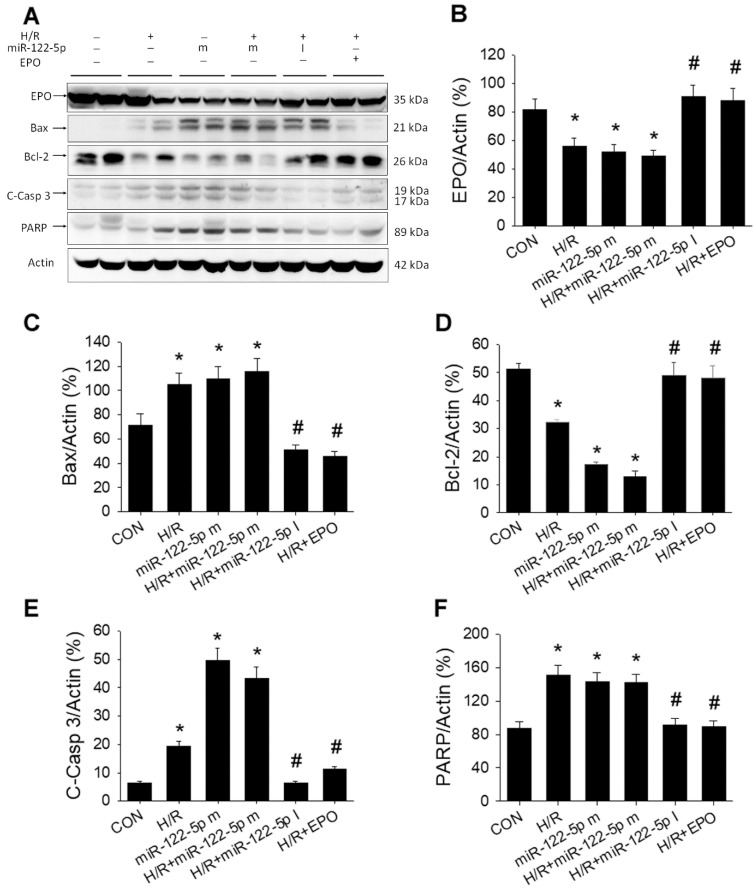
Effect of H/R, miR-122-5p mimics (m), miR-122-5p inhibitor (I), or EPO on EPO, Bax, Bcl-2, C-Casp 3, and PARP expression in NRK-52E cells by Western blot (**A**). (**B**) Statistic data of EPO expression; (**C**) statistic data of Bax expression; (**D**) statistic data of Bcl-2 expression; (**E**) statistic data of C-Caspase 3 expression; and (**F**) Statistic data of PARP expression. Data are expressed as mean ± SEM (n = 3). C-Casp 3, cleaved caspase 3; EPO, erythropoietin; H/R, hypoxia/reoxygenation; PARP, poly-(ADP-ribose)-polymerase; SEM: standard error mean. * *p* < 0.05 compared with CON group. # *p* < 0.05 compared with H/R group.

To confirm the functional role of miR-122-5p and EPO on H/R injury in the renal proximal tubular cells, we transfected miR-122-5p mimics and inhibitor into NRK-52E cells and analyzed the expression level of Bcl-2-, Bax-, C-caspase 3-, and PARP-mediated apoptosis by Western blot. As shown in Figure 8A, the original Western blot data consistently showed that H/R and/or miR-122-5p mimics markedly decreased EPO and Bcl-2 expression associated with the increased Bax, C-caspase 3, and PARP expression in NRK-52E cells. MiR-122-5p inhibitor significantly increased H/R-depressed EPO expression (*p* < 0.05, Figure 8B), Bcl-2 expression (*p* < 0.05, Figure 8D), decreased H/R enhanced Bax expression (*p* < 0.05, Figure 8C), C-Caspase 3 (*p* < 0.05, Figure 8E), and PARP expression (*p* < 0.05, Figure 8F) in NRK-52E cells. The use of EPO significantly decreased H/R injury by the preservation of EPO and Bcl-2 expression and the downregulation of Bax, C-Caspase 3, and PARP expression, subsequently leading to less apoptosis formation in the NRK-52E cells.

## 4. Discussion

Several studies utilizing mesenchymal stem cells from bone marrow, adipose tissue, and umbilical cord are ongoing, and cumulated reports of the beneficial effects of cellular therapeutics for renal IR and renal disorders are indicated [23,24,25] through the actions of paracrine effect of stem-cell-released MVs and miRNAs. In the present study, we preconditioned DEX-treated ADSC-MVs to increase more biofactors with higher migrating activity in vitro and improve renal H/R injury in vitro and IR injury in vivo. For example, DEX-treated ADSC-MVs were more efficient than ADSC-MVs at significantly increasing CD44 expression, a positive biomarker of ADSCs [15], and improve IR-depressed renal microcirculation, renal arterial blood flow, and renal function by the inhibition of apoptosis formation. We found that IR promoted the highest miR-122-5p expression among all the miRNAs in the damaged kidneys. In addition, we discovered that miR-122-5p was directly targeted to the EPO gene and downregulated EPO expression, resulting in severe apoptosis signaling in IR kidneys. We further demonstrated that the cell-free, newly, efficiently, and safely therapeutic MV from DEX preconditioning ADSCs containing specific biofactors to inhibit miR-122-5p induced detrimental effects, subsequently ameliorating renal IR injury.

Excess ROS formation contributes to renal IR injury [1,26]. ROS-induced renal proximal tubular apoptosis is a pivotal step in the pathogenesis of acute renal IR injury, which can be rescued by the treatment of antioxidant MnSOD [26]. Although strategies for inhibiting ROS production or increasing their degradation and scavenging have been attempted, the application of MV to prevent or treat renal IR injury by their ROS scavenging activity has not explored. Our present data in Figure 1F displayed that MV derived from ADSCs or DEX-preconditioned ADSC-MVs contained some antioxidant factors to directly scavenge ROS activity in vitro. Further, our in vivo model also demonstrated that both MV and DEX-MV treatments could attenuate renal IR-enhanced superoxide anion activity in the damaged kidney (Figure 2A,B). However, DEX-MVs exerted stronger antioxidant activity than MVs to decrease the ROS amount in vitro and in vivo.

One previous study reported that combined exendin-4 and ADSC therapy was superior to either one alone for improving acute kidney IR injury, suggesting the further protection raised by pharmacological preconditioning [10]. In the present study, we applied anti-inflammatory and renoprotective DEX [11,12] to precondition ADSCs. Our data found that DEX-preconditioned ADSC-MVs significantly reduced miR-122-5p-enhanced oxidative stress, Bax-/caspase 3-/PARP-mediated apoptosis, phosphorylated NF-κB-mediated inflammation, and BUN/creatinine levels in IR kidneys. Stem-cell-derived MVs or exosomes have been identified as a new mechanism of paracrine secretion of stem cells and exploited as a new therapeutic approach of stem cells to ischemic diseases [27,28]. Little is known about the biogenesis and the molecular composition of MVs produced by ADSC in different physiopathological states. Our data observed that the amount of MV protein is increased after appropriate sonication and informed that increased MV amounts could be obtained by the mechanical stimulation, whereas increased biofactors in MVs can be adapted by DEX preconditioning. Cantaluppi et al. [29] implied that MVs derived from endothelial progenitor cells protect the kidney from IR injury by delivering the miRNA cargo, which contributes to the reprogramming of hypoxic resident renal cells to a regenerative program. Our present results first implied that ADSC-MVs or DEX-preconditioned ADSC-MVs exerted renal protection against IR injury on the improvement of depressed renal microcirculation and renal blood flow and renal dysfunction. DEX-preconditioned ADSC-MVs were more efficient than ADSC-MVs in ameliorating renal hemodynamics and dysfunction through the action of downregulating miR 122-5p and negatively upregulating the target gene EPO expression.

The miRNAs regulate gene expression by inhibiting the translation process of protein-coding mRNAs through combination with the 3′ untranslated region of specific mRNAs [6,7,8,9]. After exposure to cadmium for 6 weeks, a higher expression of miR-122-5p was found in the rat kidneys, implying that its enhancement possibly contributes to renal damage [30]. According to our data, when compared to CON kidneys, a significant upregulation in miR-122-5p (↑923%), miR-142-3p (↑152%), miR-1b-5p (↑265%), miR-3068-3p (↑202%), miR-362-3p (↑167%), miR-193b-5p (↑486%), miR-1949-5p (↑551%), and miR-98-3p (↑246%), and a significant downregulation in miR-543-3p (↓73%), were found in the IR group. Our current study by microarray and quantitative PCR consistently confirmed that miR-122-5p expression was the highest expression among all the miRNAs of the IR kidneys. Our data also discovered that the elevated level of miR122-5p expression was markedly inhibited by MV or DEX-MV treatment. The decreased miR-122-5p level was more efficient in the DEXIRMV than the IRMV group. On the other hand, miR-543-3p was demonstrated in spinal cord injury for promoting locomotor function recovery and targeting several anti-inflammatory genes [31]. Our data discovered for the first time that miR-543-3p was significantly downregulated in IR kidneys and was recovered by ADSC-MVs and DEX-preconditioned ADSC-MVs (unpresented data), suggesting the beneficial role of miR-543-3p in improvement of renal injury. We further characterized these two miRNAs’ function by NRK-52E cells and evidenced the proapoptotic miR-122-5p and antiapoptotic miR-543-3p in vitro. Our unpresented data reported that upregulating miR-122-5p enhanced Bax expression, whereas upregulating miR-543-3p increased Bcl-2 and depressed Bax expression in the cultured NRK-52E cells.

IR-evoked excess oxidative stress contributed to endothelial dysfunction and decreased microcirculation and renal vasoconstriction, leading to renal dysfunction [21]. Recent evidence indicated that endothelial cells subjected to H/R injury were dysfunctional and coupled with increased apoptosis and ROS overproduction, and were rescued by endothelial progenitor cell-derived MVs via their carried miRNA-126 and eNOS activity associated with the depressed ROS production and activated PI3K/eNOS/NO pathway [32]. Our data also evidenced that ADSC-MVs or DEX treated ADSC-MVs can decrease ROS production and improve the decreased microcirculation and renal arterial blood flow, suggesting the existing antioxidant and vasodilatory factors in our prepared MVs. Importantly, our data further informed that DEX-MVs were more efficient than MVs to recover renal hemodynamics, indicating the beneficial effect of DEX pretreatment on ADSC.

As with miR-122-5p through the inhibitory action on EPO expression in the IR kidneys, our data first evidenced that upregulation in miR-122-5p could reduce EPO expression by luciferase activity and Western blot. In addition, the functional assay of miR-122-5p on Bax/Bcl-2/apoptosis signaling evidenced that miR-122-5p mimics upregulated Bax and downregulated EPO and Bcl-2 expression, consequently resulting in the increased Annexin V-apoptosis stains in the NRK-52E cells. MiR-122-5p inhibitor treatment significantly downregulated Bax and upregulated EPO and Bcl-2 expression in the NRK-52E cells. Golmohammadi et al. [33] reported that vitamin D3 and EPO protect against renal IR injury via the upregulation of heat shock protein 70 and antiapoptotic microRNA-21 expression. MiRNAs did play an important role in regulation of IR injury. Compared to mesenchymal stem-cell MVs (untreated), the EPO-treated mesenchymal stem-cell MVs had a greater benefit in reduction of apoptosis appearance in mice kidneys with unilateral ureteral obstruction, a chronic kidney disease model [34]. In response to acute kidney injury, EPO intervention can boost up the differentiation function of bone-marrow-derived mesenchymal stem cells and reverse their low secretion effect [35]. It is likely that the changes in the miRNAs’ profile and the increased EPO concentration contributed to the decreased levels of apoptosis in the proximal tubular epithelial cells, and improved renal function in kidney diseases. This would be found in our renal IR model. On the other hand, DEX preconditioning alone could ameliorate renal IR injury and inflammatory response through the p38-CD44-pathway and possibly through ischemic preconditioning [36], suggesting DEX’s effect on enhancing CD44 expression, which is a positive biomarker of rat ADSCs [15]. Exosomes or MVs of MSC origin have cell-surface markers such as CD29, CD44, and CD73 embedded in them, playing a vital role in the biomechanisms involved in the repair and regeneration, bioenergetics, immunoregulation, intracellular communication, and tissue metabolism [37]. Our data also found that intrarenal arterial DEX-MV treatment significantly increased CD44 expression compared to MV in the kidney (Figure 2C,D). The further increased CD44 expression may confer more efficient therapeutic potential in DEX-MV through the enhancement of homing and tissue-regenerating ability.

## 5. Conclusions

**Figure 9:**
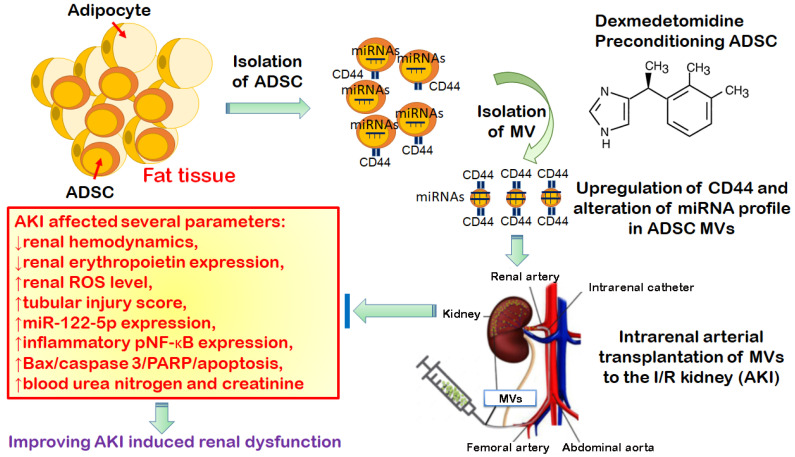
The summary diagram. The summary diagram suggests that DEX-preconditioned ADSC-derived MVs can provide CD44 homing effect; ameliorate IR-induced renal hemodynamics depression; and reduce renal ROS level, tubular cell inflammation, apoptosis, tubular injury score, and renal dysfunction through downregulated miR-122-5p/Bax/PARP/apoptosis signaling and upregulated target-gene EPO expression in the post-IR kidney. DEX-preconditioned ADSC-derived MVs could be an important tool for utilization during renal IR injury. ADSC, adipose stem cells; AKI, acute kidney injury; DEX, dexmedetomidine; EPO, erythropoietin; MV, microvesicle; I/R, ischemia/reperfusion; PARP, poly-(ADP-ribose)-polymerase; ROS, reactive oxygen species.

In conclusion, DEX-preconditioned ADSC-derived MVs can ameliorate IR-induced tubular cell apoptosis and renal dysfunction through downregulated miR-122-5p/Bax/PARP/apoptosis signaling and upregulated target-gene EPO expression in the post-IR kidney (Figure 9). DEX-preconditioned ADSC-derived MVs could be an important tool for utilization during renal IR injury. Furthermore, an actual advantage would come from the therapeutic use of MVs with respect to MSCs, avoiding the possible immune rejection and lung entrapment, improving the safety, and allowing the crossing of biological barriers.

## Data Availability

The data presented in this study are available on request from the corresponding author.

## Abbreviation

ADSC	adipose-derived stem cells;
BSA	bovine serum albumin;
DEXMV	dexmedetomidine preconditioning adipose-derived stem cell microvesicles;
DMEM	Dulbecco’s modified Eagle’s medium;
EPO	erythropoietin;
FBS	fetal bovine serum;
H/R	hypoxia/reoxygenation;
IRDEXMV	ischemia/reperfusion and dexmedetomidine preconditioning adipose-derived stem cell microvesicles;
IRMV	ischemia/reperfusion and adipose-derived stem cell microvesicles;
IR	ischemia/reperfusion;
miRNA	microRNA;
MTT	3-(4,5-Dimethylthiazol-2-yl)-2,5-diphenyltetrazolium bromide;
MV	microvesicles;
ROS	reactive oxygen species;
SVF	stromal vascular fraction;
NF-κB	nuclear factor-kappa B;
PARP	poly(ADP-ribose) polymerase
